# Comparison of gut viral communities in children under 5 years old and newborns

**DOI:** 10.1186/s12985-023-02013-2

**Published:** 2023-03-27

**Authors:** Hong Li, Hao Wang, Huimin Ju, Jinquan Lv, Shixing Yang, Wen Zhang, Hongyan Lu

**Affiliations:** 1 Department of Pediatrics, Affiliated Hospital of Jiangsu University, Zhenjiang, Jiangsu 212000 P.R. China; 2 School of Medicine, Jiangsu University, Zhenjiang, Jiangsu 212013 China; 3 Department of Clinical Laboratory, Huai’an Hospital, Xuzhou Medical University, Huai’an, Jiangsu 223002 China

**Keywords:** Children, Newborn, Viral metagenomics, Bacteriophage, Virome, Gut viral communities

## Abstract

**Objectives:**

The gut virome of humans is mainly composed of bacteriophages and their role in shaping the gut microbiome and influencing human health is increasingly recognized. However, little is known about the dynamic changes of the gut virome in children and its role in growth and development. In this study, we collected fecal samples from newborns and children under 5 years old from the same area during the same time period to investigate the gut viral community using viral metagenomic technique.

**Methods:**

We used viral metagenomics to compare the gut bacteriophage composition between newborns and children under 5 years of age. We collected fecal samples from 45 newborns who were born at the Affiliated Hospital of Jiangsu University and 45 healthy children who were examined at the same hospital. The two groups were classified as the newborn group and the children group.

**Results:**

Our sequencing analysis showed that the number of seqeunce reads of the children group were more than that of the newborn group. The results of alpha diversity and beta diversity both indicated that the diversity of the children group was significantly higher than that of the newborn group and the children group is different from the newborn group. The abundance of gut virome in the children group was also higher than that in the newborn group. The analysis of the genetic characteristics of the viruses showed that the phage genome was scattered and clustered with specificity.

**Conclusion:**

Our findings indicate that the gut bacteriophage communities undergo changes over time, presenting diversity and dynamic characteristics. We characterized the composition of gut virome in children and newborns in this region. However, further research is needed to investigate the function of bacteriophages in the ecology of the gastrointestinal tract.

## Introduction

According to the estimation of causes of death in children younger than 5 years old and newborn by WHO, pneumonia, diarrhoea, malaria, neonatal pneumonia or sepsis account for more than half of all child deaths, which are caused by infection of bacteria, fungi and viruses[1–5]. Factors such as genetics, diet, environment, and immunity can affect children’s health. Recent research has shown that the intestinal microbiome plays an important role in the growth, development, and health of children. Gut microbiota is associated with many diseases, including childhood growth and development, diabetes, inflammatory bowel disease and obesity [6–9]. Phages can affect the diversity and abundance of intestinal bacteria [10, 11], so they may be involved in host metabolism and immune regulation. Currently, little is known about the role that phages play in child health and the distribution of phages at different ages.

The gut of healthy human newborn is usually virus-free, but it can be infected by virus quickly [12]. During the early years of children, the viral microbiome of their guts is less known, including bacteriophages and eukaryotic RNA and DNA viruses [13]. It has been reported that bacteriophages can drive evolutionary changes in bacterial communities by creating gene-flow networks that promote ecological adaptation [14]. The gut bacteriophages exhibit a high degree of diversity and dynamic during the first few months of life and gradually decrease over time [15]. One study showed that the gut microbiomes of twins were more similar than those of unrelated individuals [16]. Therefore, the gut microbiome is also associated with a variety of factors. Ultimately, the distribution of intestinal phages varies according to the individual’s health [17]. Mammalian gut bacteriophages mainly include double-stranded DNA (dsDNA) belonging to *Caudovirales*, represented by the *Siphoviridae*, *Podoviridae* and *Myoviridae* families and single-stranded DNA (ssDNA) belonging to *Microviridae* family [18, 19].

In the current study, we analysed the gut bacteriophage communities in children under 5 years old and newborn by using viral metagenomics to understand the diversity and dynamics of bacteriophages, and map the distribution of intestinal phages in a part of healthy children.

## Materials and Methods

### Sample collection and preparation

To investigate the gut bacteriophage composition between newborn and children under 5 years of age, 90 fecal specimens were respectively collected from 45 newborn who were born in the Affiliated Hospital of Jiangsu University (Zhenjiang City, Jiangsu Province, China)and 45 healthy children who were examined at the same hospital from Sep. to Dec. 2018. All samples were collected with 1.5ml disposable sterile tubes and transported to the laboratory via dry ice, and then stored in the refrigerator at -80℃. The specimens were collected with the written consent of the guardian. Each fecal specimen was added ten volumes of Dulbecco’s Phosphate Buffered Saline (DPBS). Each specimen was vigorously vortexed for 5 min and it was repeated for three times. The supernatants were collected after centrifugation (10 min, 15,000 × g), then the specimens were stored in the refrigerator at -80℃ until the viral metagenomic analysis was performed. Sample collection and all experiments in the present study were performed with Ethical Approval given by Ethics Committee of Jiangsu University and the reference number is No. UJS2018030.

### Viral metagenomic analysis

Prepared supernatants from 45 newborn and 45 children were respectively pooled into sample pools according to the age group, 90 sample supernatants were pooled into 10 pools with 9-sample each. Supernatant pools were filtered through a 0.45 μm filter (Millipore) to remove eukaryotic- and bacterial cell-sized particles, and 200 µL of supernatant from each pool was then subjected to a mixture of nuclease enzymes to reduce the concentration of free (non-viral encapsidated) nucleic acids. The filtrates enriched in viral particles were treated with DNase and RNase to digest unprotected nucleic acid at 37℃ for 90 min [20, 21].

Total viral nucleic acids were extracted according to the procedures of QIAamp Viral MinElute Virus Spin Kit (Qiagen). Then viral nucleic acid was reversed into double-stranded DNA using SuperScript III Reverse transcriptase kit (Invitgen) and Klenow Enzyme (NEB) according to the manufacture’s protocols. 10 libraries were then constructed using Nextera XT DNA Sample Preparation Kit (Illumina) and sequenced using the MiSeq Illumina platform with 250 bp paired ends with dual barcoding for each pool [22].

### Bioinformatics analysis

For bioinformatics analysis, paired-end reads of 250 bp generated by MiSeq were debarcoded using vendor software from Illumina. An in-house analysis pipeline running on a 32-nodes Linux cluster was used to process the data. Clonal reads were removed and low sequencing quality tails were trimmed using Phred quality score 20 as the threshold. Adaptors were trimmed using the default parameters of VecScreen which is NCBI BLASTn with specialized parameters designed for adapter removal. The cleaned reads were de novo assembled by SOAPdenovo2 version r240 using Kmer size 63 with default settings. The assembled contigs along with singlets were matched to an in-house viral proteome database using BLASTx with an E-value cutoff of < 10^− 5^ [23, 24]. These BLASTx results generated by DIAMOND (DAA format) were used to generate rma6 format files by MEGAN6 software, which can be further used for subsequent analysis including species accumulation curve, and Co-occurrence plot [25].

### Phylogenetic analysis

Phylogenetic analysis was performed based on predicted viral amino acid sequences and their closest viral relatives on the best BLASTp hits in GenBank and representative members of related viral species or genera. CLUSTAL X (version 2.1) was used to perform the sequence alignment with default settings and phylogenetic trees were generated by Bayesian Inference (BI) in MrBayes 3.2 [26].

### Statistical analysis

To compare differences in viral diversity between groups, statistical analyses were performed using MEGAN6 and R version 4.0.3 Alpha diversity and beta diversity were performed using the vegan package and Wilcoxon tests was used for two-group comparisons, respectively. There was considered statistically significant when *p* < 0.05. The Comparison of virus alpha diversity was demonstrated by Shannon index analysis. The Beta diversity analysis based on Bray-Curtis included Analysis of similarities, Principal coordinate analysis and the Unweighted Pair-group Method with Arithmetic averages [27].

## Results

### Overview of sequencing data

In the study, to facilitate the comparison of gut bacteriophage communities in children under 5 years of age and newborn, the study cohort were divided into children and newborn groups.

A total of 3,462,741 raw reads were obtained from these 10 libraries including 5 children pools and 5 newborn pools based on next generation sequencing. 55,741 reads were related to bacteriophages according to the de novo assembled and compared with the GenBank non-redundant protein database through BLASTx. The number of sequence reads of the children group were more than that of the newborn group. Detailed information of sequence read numbers were displayed in the Table 1. Sequence de novo assembly generated 26 complete genomic of the family *Microviridae* and with sequence length ranges from 4458 to 7718. The other incomplete sequences with complete open reading frame (ORF) of the major capsid proteins were also obtained.

**Table 1:** Raw reads and bacteriophages-related reads of each library

Library No.	Raw reads	Bacteriophages related reads	Library No.	Raw reads	Bacteriophages related reads
Children01	536,357	9617	Newborn01	124,266	232
Children02	291,565	13,991	Newborn02	138,671	3438
Children03	1,051,185	13,822	Newborn03	199,378	2230
Children04	517,793	4145	Newborn04	237,704	4637
Children05	300,505	2598	Newborn05	65,317	1031

### Comparison of gut bacteriophage communities

**Fig. 1:**
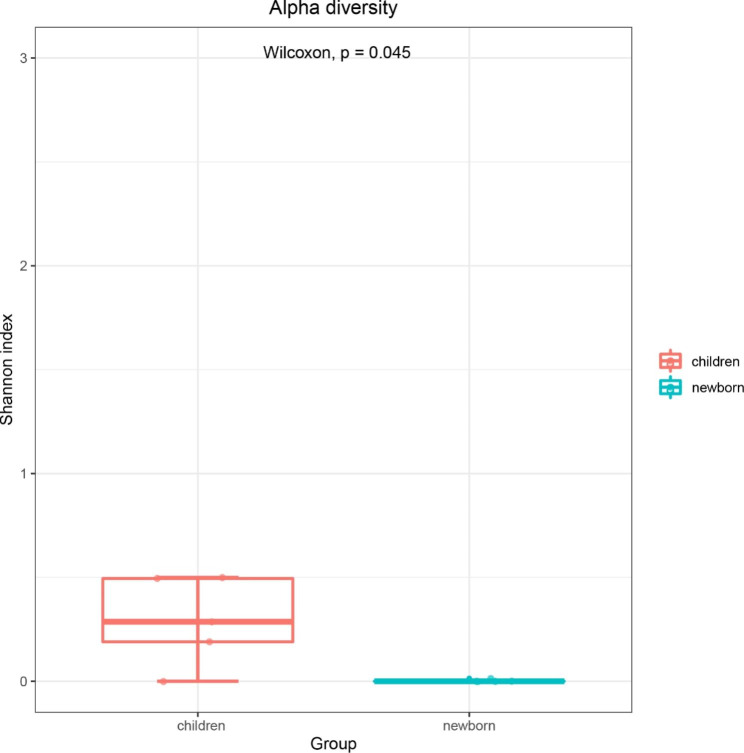
Alpha diversity analysis. Comparison of virus alpha diversity (Shannon index) between the children group and the newborn group

**Fig. 2:**
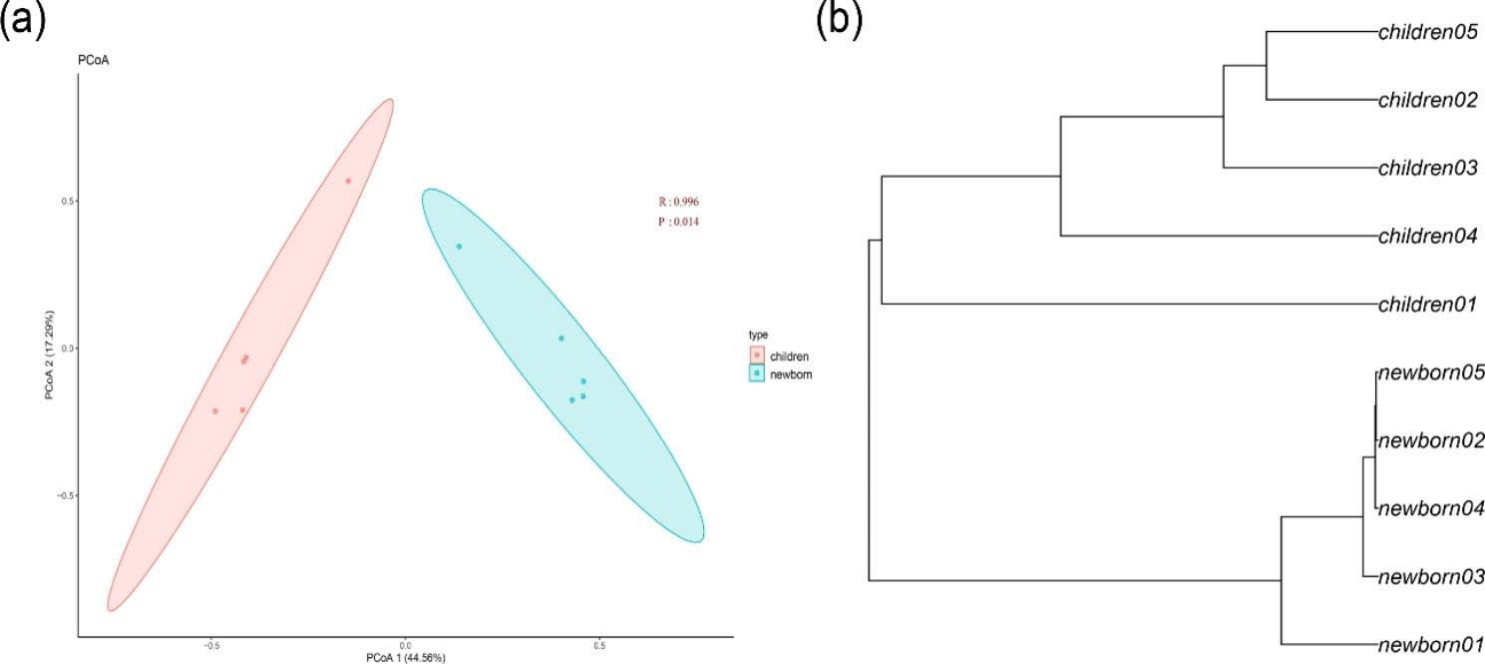
Beta diversity analysis. (a) Principal coordinate analysis (PCoA) scatter plot. Circles represent the 95% normal probability ellipse for each group. The pink one was on behalf of the children group and the blue one was on behalf of the newborn group. (b) The hierarchical clustering tree based on Bray–Curtis and built with the Unweighted Pair-group Method with Arithmetic averages (UPGMA).

In the family level, alpha diversity was used to show the difference of gut bacteriophage community composition between the children and the newborn group where the Shannon index indicated that the diversity of the children group was significantly higher than that of the newborn group (p = 0.045, Wilcoxon test) (Fig. 1). Based on beta diversity, unweighted UniFrac analysis suggested that principal coordinate analysis (PCoA) (Fig. 2a) and hierarchical clustering can distinguish the children group from the newborn group (Fig. 2b).

**Fig. 3:**
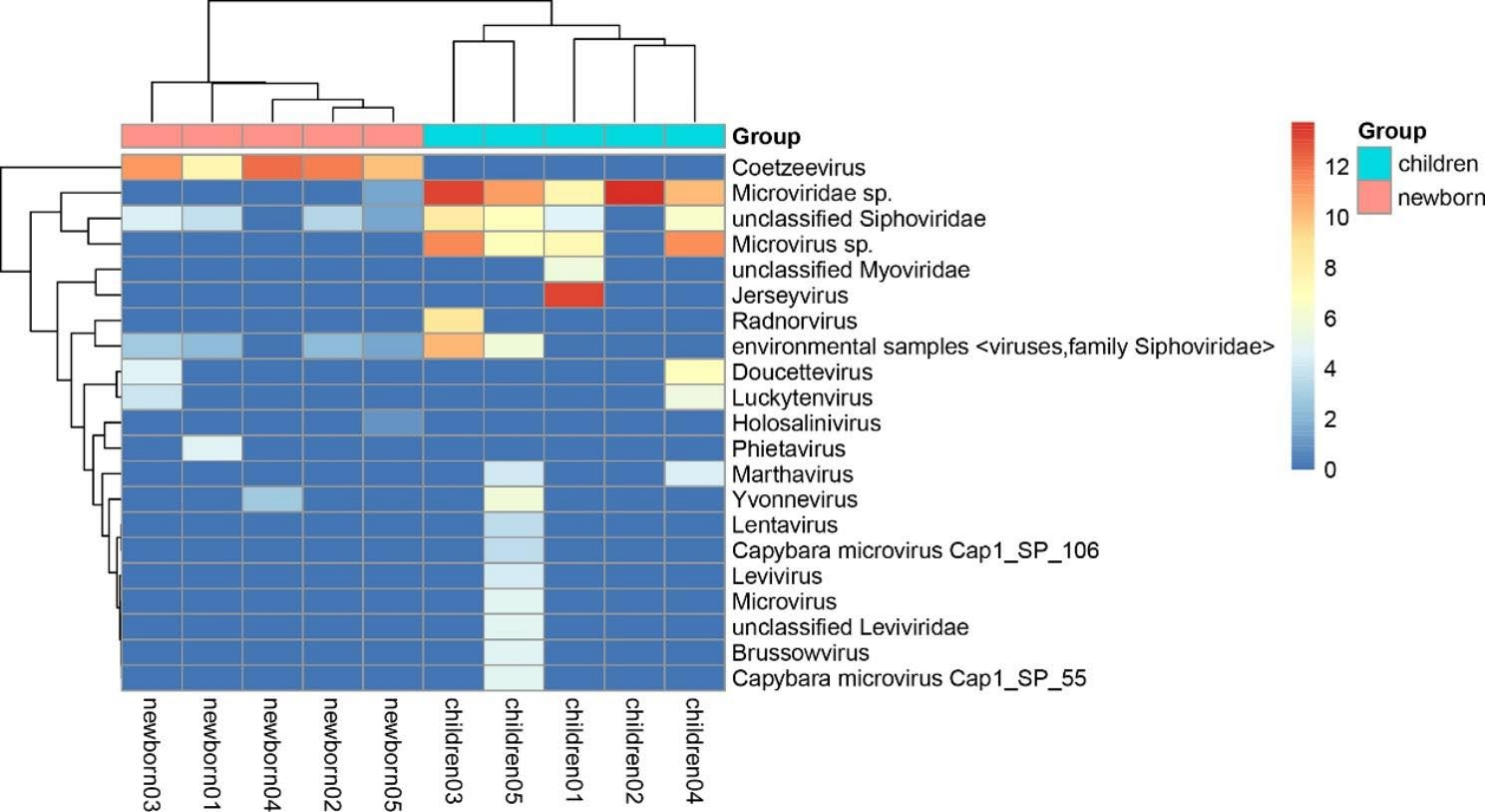
Comparison of gut bacteriophage communities between the children group and the newborn group. Clustering heatmap of representative viruses from the 10 libraries. The bottom of the figure represents the library number. The red bar at the top of the figure represents the newborn group and the blue bar represents the children group. The right of the figure is the name of representative viruses. The number of reads is logarithmically converted with log10 as the base which is shown in the upper right corner

A great difference of the gut bacteriophage communities between the children group and newborn group were observed. Coetzeevirus was detected in the newborn group, but not in the children group. In the children group, the number of viral reads such as family *Microviridae*, *Siphoviridae* and *Myoviridae* were more than those in the newborn group, where family *Microviridae* was detected in all children libraries and *Siphoviridae* was abundant in all children groups except the children02 (Fig. 3).

### Phylogenetic analysis of main gut bacteriophages detected in the two groups

To further assess the diversity of the gut bacteriophage communities of the children and newborn groups, phylogenetic analyses were performed based on the signature amino acid sequences of the main gut bacteriophages acquired in this study and reference sequences from GenBank, including the major capsid protein for *Microviridae* and phage terminase large subunit (TerL) for *Siphoviridae* and *Myoviridae*, respectively.

**Fig. 4:**
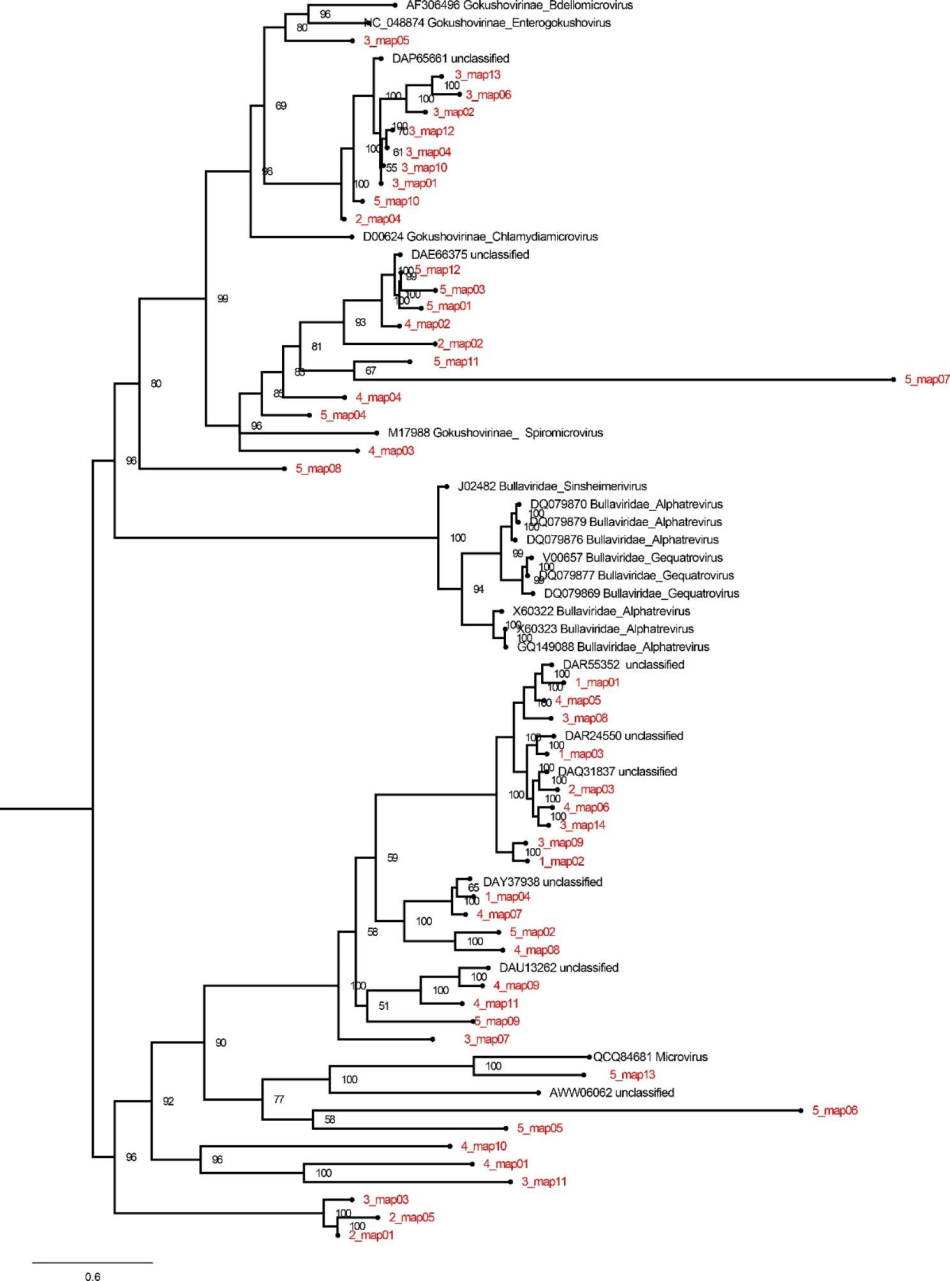
Phylogenetic analysis was performed on the amino acid sequences of the major capsid protein of the family *Microviridae* in the children group. The identified sequences in this study were labeled in red

In this study, amino acid sequences of the major capsid protein of the family *Microviridae* in the children group were used to construct the phylogenetic tree and assess the microviral diversity. The result indicated that most of these sequences were clustered divergently and formed several branches (Fig. 4).

**Fig. 5:**
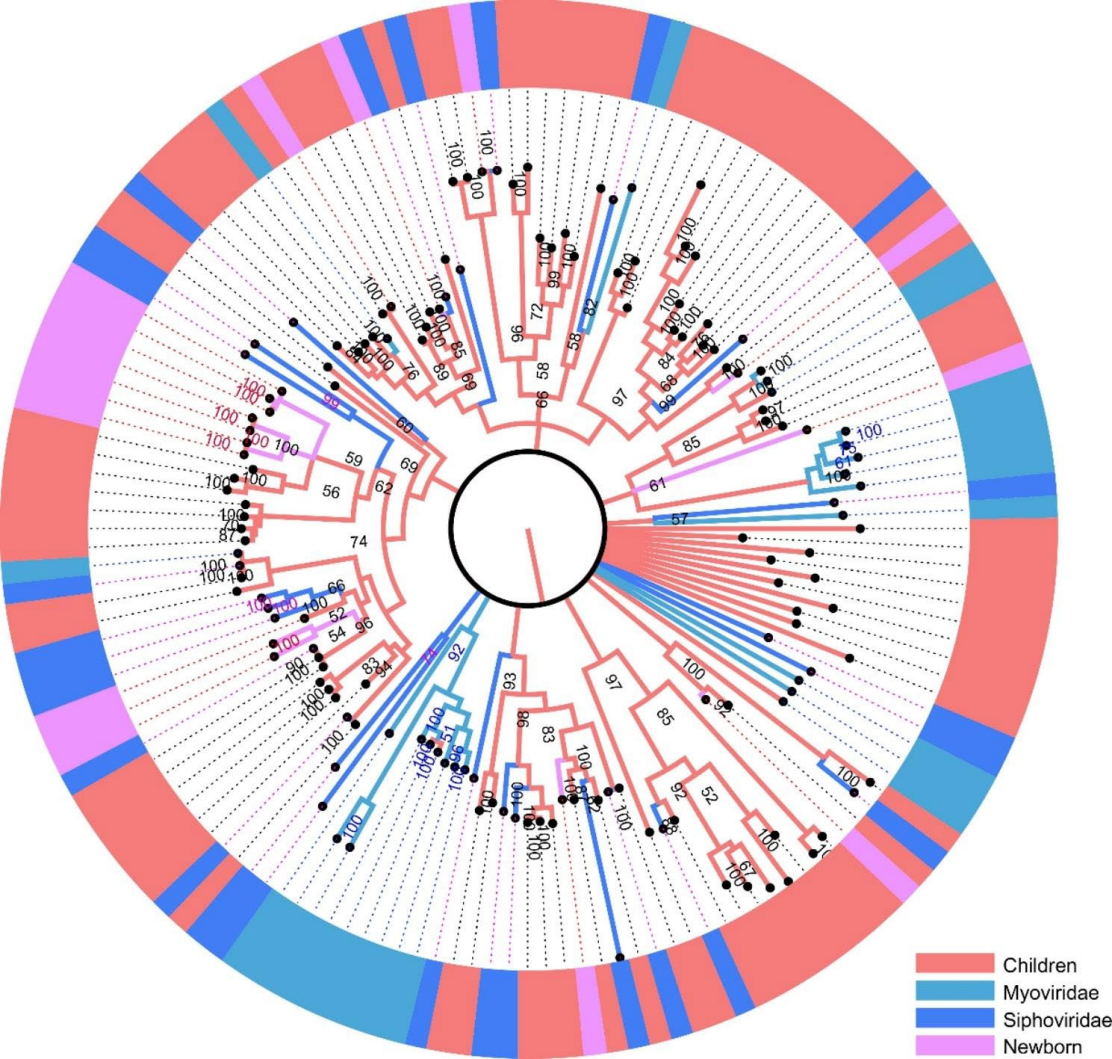
Phylogenetic analysis was performed on the amino acid sequences of the family *Siphoviridae* and *Myoviridae* in the children group and the newborn group. The viruses found in the children group was labeled in red and the newborn group was labeled in pink. Reference sequences and corresponding viruses are labeled with consistent colors

The family *Siphoviridae* and *Myoviridae* belongs to the dsDNA bacteriophage with a conserved region known as terminase large subunit (TerL). A phylogenetic tree was constructed based on the TerL sequences of the family *Siphoviridae* and *Myoviridae* identified in both children and newborn groups. The topological structure of the tree suggested that most of these sequences were too divergent to be classified into known family of *Siphoviridae* and *Myoviridae* and several new clades were formed (Fig. 5), suggesting putative novel phage families present in the gut of children and newborns. The number of hallmark gene sequences assembled from children group is much more than that assembled from the newborn group, which is consisted with the above results that gut phage diversity in the children is higher than that in the newborn. Our data also indicated that sequences of the family *Siphoviridae* from the newborn group were clustered with sequences from the children group (Fig. 5), implying phage communities from gut of children and newborns share the common species of siphovirus.

## Discussion

Viral metagenomics is a widely used tool to discover new viruses, map viral genomes, and investigate viral diversity. In the past decade, with the rapid development of sequencing technology and bioinformatics, metagenomics has enabled detailed exploration of the infant gut microbiome, including its diversity in various pathological conditions such as childhood developmental delays and food allergies [6, 28].

In this study, we utilized viral metagenomics to analyze the gut bacteriophage communities in children under 5 years old and newborns, aiming to understand the diversity and dynamics of bacteriophages. Our sequencing analysis revealed that the children group had more reads than the newborn group. Furthermore, both α-diversity and β-diversity analyses showed that the intestinal phage diversity in children was significantly higher than that in newborns. This may be due to the fact that newborns only consume milk or breast milk, and are in a protected environment that does not yet establish a stable intestinal phage community. In our hierarchical clustering analysis, we detected Coetzeevirus in the library of the newborn group but not in the library of the children group, which may be related to early neonatal exposure. Coetzeevirus belongs to the Lactobacillus phage group [29], and our survey indicated that newborns in China are frequently given Bifidobacterium, which could explain the high abundance of Coetzeevirus in the newborn group. In contrast, Yvonnevirus, Lentarirus, Brussowvirus, Capybara microvirus, and Leviviridae were detected in the children05 library, which could be associated with DNA amplification bias or different best matches in BLASTx (E-value cutoff of < 10^− 5^).

Comparing the two groups, the children group had more viral reads from families such as *Microviridae*, *Siphoviridae*, and *Myoviridae* than the newborn group. A previous study on intestinal phages in healthy adults found mainly *Microviridae*, *Myoviridae*, *Siphoviridae*, and Unassigned at the family level [30]. In our complete genome analysis, we obtained 26 complete genomes of *Microviridae*, suggesting that *Microviridae* is abundant in children. However, we lack comprehensive longitudinal observation for the two groups of subjects, so there is no specific timeline for the children group to form the current distribution state of gut virome. Myoviridae and Siphoviridae are members of the Caudovirales family, sharing a central tape measure protein surrounded by a tail tube and ending with a terminator protein. [31, 32]. The phylogenetic analyses performed on the major capsid protein of the family *Microviridae* and phage terminase large subunit (TerL) of the family *Siphoviridae* and *Myoviridae* indicated that most of these sequences detected in this study were clustered divergently and formed several branches. Studies have shown that the composition of adult gut virome appears to be highly specific and stable. Most phages appear unique to everyone [16, 33, 34]. The variation could have led to this result and could also explain the scattered clustering of the sequences detected in our experiment.

Clearly, there are some limitations to our experiment. First, the sample size of this study is insufficient, resulting in incomplete results. However, it also provides a certain value for the study of gut virome in newborn and children. Second, the geographical area of sample collection is too narrow. China is a large country with abundant resources, and there are great differences in climate and dietary habits among different regions, which also leads to great differences in the distribution of environmental virus species in different regions. These factors may lead to some different gut virome in children, but this needs to be confirmed by follow-up experiments. Third, compared with the study of intestinal bacterial spectrum, there are relatively few studies on gut virome, which also leads to the lack of data in relevant databases at present, so that our data analysis is limited to a certain extent.

## Conclusions

It is evident that our experiment has some limitations. Firstly, the sample size of this study is insufficient, resulting in incomplete findings. However, it does offer some value for exploring the gut virome in newborns and children. Secondly, the geographic area where we collected samples is too limited. China is a vast country with diverse resources, and there are significant variations in climate and dietary habits across different regions, resulting in different distributions of environmental virus species. These factors could lead to distinct gut viromes in children, but further research is needed to confirm this. Thirdly, compared to the study of intestinal bacterial spectrum, there are relatively few investigations on the gut virome, leading to a lack of data in relevant databases currently, which limits our data analysis to some extent.

## Data Availability

The raw sequence reads generated from samples of newborn and heathy children were deposited into the Sequence Read Archive of GenBank database and the accession nos. are SRX17239726, SRX17239090, SRX17239110, SRX17239718, SRX17239522, SRX17171841, SRX17206676, SRX17208350, SRX17206782, and SRX17206885. This information is available free of charge on the ACS Publications website.

## Abbreviations

TerL: Terminase large subunit
WHO: The World Health Organization website
dsDNA: Double-stranded DNA
ssDNA: Single-stranded DNA
DPBS: Dulbecco’s Phosphate Buffered Saline
PCR: Polymerase chain reaction
NCBI: The National Center for Biotechnology Information
BI: Bayesian Inference
PCoA: Principal coordinate analysis
